# Tuberculosis of the Urinary System Treated by Tuberculin

**Published:** 1906-01-27

**Authors:** 


### TUBERCULOSIS OF THE URINARY SYSTEM TREATED BY TUBERCULIN.

THESE columns recently contained an abstracted account of a series of cases of chronic pulmonary tuberculosis, in the treatment of which the new tuberculin (T.R.) was employed, subject to the control of observations upon the "opsonic index" of the blood. At a subsequent meeting of the Medico-Chirurgical Society [1] a further communication on this method of treating tuberculosis was made by Mr. John Pardoe, the lesions in this instance being those of the urinary system. This author contends that primary tuberculosis of the urinary bladder is of far more frequent occurrence than is generally recognised: that the large majority of such cases generally drift, as cases of "obstinate cystitis," into an advanced stage before a correct diagnosis is made. "The treatment of tuberculosis of the urinary tract (particularly the bladder) by operative measures is so disappointing in my experience that I have almost ceased to employ them," he says, "whilst tuberculosis of the kidneys is usually so far advanced, and so often accompanied by infection of other parts of the urinary tract when operations are resorted to, that permanent benefit seldom seems to result."

The lesion in cases of tuberculosis of the urinary bladder is almost never a solitary one: consequently excision is generally impracticable; and short of excision, the local treatment of the condition has generally resolved itself into simple suprapubic drainage, or the injection of various emulsions and solutions containing drugs such as iodoform or salts of copper and mercury. The poor results obtained by such means led Mr. Pardoe to attempt the use of tuberculin (T.R.). The nature of this vaccine was explained in our previous reference to this subject: Mr. Pardoe has employed it as it is sold, with the exception that he is in the habit of sterilising the tuberculin by an exposure to a temperature of 60° C. for one hour. This procedure has been shown to be necessary by the experiments of Thellung, who was able to infect rabbits and guinea-pigs by injections of the tuberculin, a result which demonstrated that the latter occasionally contained living and active bacilli. Wright and Douglas have shown that exposure to the temperature above named does not injuriously affect the activity of tuberculin.

Mr. Pardoe describes the effect of tuberculin injections as follows:—

1. *At the site of injection.*—In the majority of cases there is very little local effect, though occasionally a little swelling and hardness appear around the puncture. But on rare occasions a marked local reaction may be observed, especially with large doses.

2. *Effect upon tuberculous deposits.*—The effect of the old tuberculin upon tuberculous deposits consisted of a vigorous reaction round the affected areas, evidenced by swelling, heat, and redness, and followed by necrosis and sloughing of the diseased tissues. It is said that these effects are not associated with tuberculin (T.R.), but Mr. Pardoe's experience in cases of tuberculosis of the urinary bladder does not corroborate this statement. When anything like a large dose was employed there followed an increase of pain on micturition, hæmaturia and pyuria, with general malaise, and occasionally a sharp rise of temperature. He is of opinion that these symptoms are certainly to be credited to active changes occurring in the neighbourhood of tuberculous areas. Confirmatory evidence on this point was afforded by a cystoscopy undertaken during one of these exacerbations. It was then seen that the discrete tubercles previously observed were surrounded by a wider-spread zone of hyperæmia and congestion, the bladder capacity at the same time being reduced by about one-half. This phase passed away on diminishing the dose and lengthening the intervals between the injections.

3. *Effect upon the system.*—Wright and Douglas consider that the characteristic feature of a tubercle vaccine is the presence in it of "a derivative of the protoplasm of the tubercle bacillus, which is capable of producing an elaboration of tuberculo-tropic substances in the organism." This hypothesis led to the invocation of opsonic indices, the nature of which has been lately dealt with in these columns The particular point of interest distinguishing the work under notice from that on which our last comments were made, lies in the fact that in the series of cases considered by Mr. Pardoe the dosage was not regulated by any consideration of the opsonic state of the blood, but by clinical observation of the bodily state of the patient. Mr. Pardoe admits that if the existence and mensuration of opsonins become indisputable facts, then clinicians may need to revise their methods of administering tuberculin. But till then the fact that the estimation of opsonic curves requires the assistance of a skilled bacteriologist, and an immense sacrifice of time, must relegate such estimations to a sphere outside that of ordinary therapeutics. He considers that it is usually obvious from the patient's condition when the maximum dose to be used with advantage has been reached, and that symptoms of overdose are never met with until the dose exceeds one two-hundredth of a milligramme.

The author urges as an important point of practice that all cases of urinary tuberculosis are not suitable for treatment by tuberculin, simply on account of the reaction likely to be set up by the use of this substance. When both ureters are known to be affected, or where presumption points in this direction, the use of tuberculin may lead to such a degree of bilateral swelling of the ureters as will completely obliterate their lumen and set up a fatal suppression of urine: as indeed happened in one of the cases of Mr. Pardoe's series. He urges, therefore, that the tuberculin treatment of urinary tuberculosis should never be begun until a cystoscopic examination has rendered it fairly certain that one ureter at least is discharging clear urine, and is not obviously affected.

The method employed by this author is to administer the tuberculin subcutaneously with a sterilised syringe. The commencing dose is one five-hundredth of a milligramme, this dose being gradually increased, and injections administered every other day, until a definite reaction is obtained. This usually consists of a rise of temperature by two or three degrees, accompanied by some malaise and increase of pain upon micturition. The dose is then reduced to that which causes no apparent reaction, and is given steadily once a week for long periods. Should a further definite reaction occur the dose is once more diminished and given at longer intervals.

Mr. Pardoe is particular to qualify his adhesion to clinical methods of estimating the dosage of tuberculin by the observation that in cases of tuberculosis of the urinary system the evidence of overdose is delicate, and the response too obvious to be overlooked. He admits that in lesions of other parts of the body opsonic estimations may be absolutely essential.

The author's paper is founded upon a series of 21 patients, all of whom have been under observation for upwards of a year. Of these patients six have died, six remain *in statu quo*, four are much improved, and five appear to be cured entirely.

The main conclusions drawn by Mr. Pardoe are as follows:—That other methods of treating tuberculosis of the urinary tract are disappointing in the extreme, for he himself has never seen an apparent cure of vesical tuberculosis by operation, and the published records of such cases are very few. That the earlier the treatment is begun, the greater is the likelihood of cure. That in T.R. we have not a perfect remedy for tuberculosis of the urinary system, but that it is the best at our disposal; for whereas without it there is little hope of effecting much improvement, with it there is the hope, in some cases of cure, and in a far larger number of great improvement.

[1] Medico-Chir. Soc., Dec. 12th, 1905.

